# Developing a novel mobile application for cognitive behavioral therapy for insomnia for people with schizophrenia: integration of wearable and environmental sleep sensors

**DOI:** 10.1007/s11325-023-02980-4

**Published:** 2024-01-04

**Authors:** Jae Min Jeon, Junhua Ma, Paulyn Kwak, Bing Dang, Italo Buleje, Sonia Ancoli-Israel, Atul Malhotra, Ellen E. Lee

**Affiliations:** 1 Department of Psychiatry, University of California San Diego, 9500 Gilman Dr #0664, La Jolla, CA 92093-0664, USA; 2 Digital Health, IBM T.J. Watson Research Center, 1101 Kitchawan Rd, Yorktown Heights, NY 10598, USA; 3 Division of Pulmonary, Critical Care and Sleep Medicine, University of California San Diego, 4520 Executive Dr Suite P2, San Diego, CA 92121, USA; 4 Desert-Pacific Mental Illness Research Education and Clinical Center, Veterans Affairs San Diego Healthcare System, 3350 La Jolla Village Dr, San Diego, CA 92161, USA

**Keywords:** Digital health technology, Application development, Sensors

## Abstract

**Background:**

People with serious mental illnesses (SMIs) have three-fold higher rates of comorbid insomnia than the general population, which has downstream effects on cognitive, mental, and physical health. Cognitive Behavioral Therapy for Insomnia (CBT-i) is a safe and effective first-line treatment for insomnia, though the therapy’s effectiveness relies on completing nightly sleep diaries which can be challenging for some people with SMI and comorbid cognitive deficits. Supportive technologies such as mobile applications and sleep sensors may aid with completing sleep diaries. However, commercially available CBT-i apps are not designed for individuals with cognitive deficits. To aid with this challenge, we have developed an integrated mobile application, named “Sleep Catcher,” that will automatically incorporate data from a wearable fitness tracker and a bed sensor to track nightly sleep duration, overnight awakenings, bed-times, and wake-times to generate nightly sleep diaries for CBT-i.

**Methods:**

The application development process will be described—writing algorithms to generating useful data, creating a clinician web portal to oversee patients and the mobile application, and integrating sleep data from device platforms and user input.

**Results:**

The mobile and web applications were developed using Flutter, IBM Code Engine, and IBM Cloudant database. The mobile application was developed with a user-centered approach and incremental changes informed by a series of beta tests. Special user-interface features were considered to address the challenges of developing a simple and effective mobile application targeting people with SMI.

**Conclusion:**

There is strong potential for synergy between engineering and mental health expertise to develop technologies for specific clinical populations. Digital health technologies allow for the development of multi-disciplinary solutions to existing health disparities in vulnerable populations, particularly in people with SMI.

## Introduction

Nearly a third of patients with psychiatric disorders meet criteria for insomnia [1]. Treatment of insomnia has the potential to improve mental health and physical health outcomes [1, 2] for people with depression [3], schizophrenia [4], bipolar disorder [5], and post-traumatic stress disorder [6]. Cognitive Behavioral Therapy for Insomnia (CBT-i) is the first-line treatment for insomnia due to its sustained benefits and low risk of adverse side effects compared to pharmacotherapy [7]. Six-week CBT-i aims to provide psychoeducation about sleep, modify perception of poor sleep, and improve habits that affect sleep. Individuals undergoing CBT-i are instructed to keep a daily sleep diary that the therapist uses to formulate a plan to change sleep behaviors. For example, when a patient reports frequent overnight awakenings and difficulty falling back asleep in the nightly diary, the therapist will tailor their recommendations to reduce time spent lying in bed while awake.

Studies have shown the beneficial effects of CBT-i for psychiatric populations [6], though studies including patients with serious mental illnesses (SMI), such as schizophrenia or bipolar disorder, are limited [8–11]. Despite the benefits of CBT-i and the lack of associated side effects, one major barrier to successful CBT-i is the lack of adherence to the sleep diaries, which informs the specific changes for each individual [12]. In particular, individuals with SMI may have challenges in completing the nightly sleep diaries due to irregular routines and working memory deficits [12, 13]. In addition, commercially available CBT-i mobile applications (e.g., CBT-i Coach, Sleepio, Shuteye, and Somyrst) include tools to improve their sleep though their usability for clinical populations may be limited. CBT-i Coach, a free mobile application, allows users to complete sleep diary entries but lacks an updated user interface and clear instructions on how it may be used clinically. Applications requiring purchases (e.g., Sleepio, Shuteye, and Somyrst) include tools and resources for clinical application, but they are located behind the paywall and may be inaccessible for some users. While insurance companies may cover these health-related costs, users would have to seek out the referrals needed for coverage. Additionally, all these applications lack separate sleep sensor integration, though the data from sleep trackers could aid users with sleep diary completion, a crucial component of CBT-i.

To support CBT-i for people with SMI and cognitive impairments, we aimed to develop a mobile application, named “Sleep Catcher,” with integration of sleep sensors that will automatically and accurately collect participant’s sleep data and support completion of sleep diaries.

## Methods

### Application principles and devices

To simplify CBT-i for people with SMI, our application focused on the four cardinal rules for brief behavioral therapy for insomnia [14]: (1) reduce your time spent in bed while awake, (2) wake up at the same time every day, (3) do not get into bed unless you are sleepy, and (4) do not stay in bed unless you are asleep. The sleep diaries are used to guide the therapist’s recommendations around these rules.

Our mobile application leverages two commercially available sleep trackers to generate sleep diaries automatically. Data from the wrist-worn Fitbit Charge 5 fitness tracker and Withings sleep mat are used to complete nightly sleep diary entries: get into bed time, fall asleep time, wake up time, get out of bed time, number of awakenings, and time spent awake. These devices were chosen for their strengths in Web Application Programming Interface (API), which enabled robust integration into our application. The Fitbit device assesses movement and heart rate patterns, providing reliable detection of when the user falls asleep and wakes up [15]. The Withings device has a pneumatic sensor to detect movements while in bed and sleep, providing accurate assessment of when the user gets into bed and gets out of bed [16].

### Agile methodology

We used the Agile methodology for software development, a popular and effective approach that prioritizes the customer’s needs while allowing flexibility, adaptability to changes, and continual improvement. The project team included both engineers and mental health researchers to integrate their expertise and co-develop the application for people with SMI and associated cognitive deficits. The *requirement gathering phase* is necessary to gain insight about the target user population (i.e., people with SMI) and clearly identify specific problems to solve via the application. The engineering team shadowed research visits with community-dwelling participants with SMI from San Diego County. Many research participants had some degree of cognitive impairment, did not own a smartphone, and lacked familiarity with wearable sleep sensors. These findings emphasized the need for simplicity in our application design for the users. In line with *sprints during development*, the engineering team provided weekly progress reports to the psychiatry team, receiving immediate feedback on newly developed features and improvements. The *communication* principle aided efficient collaboration between the engineering and psychiatry teams, keeping the development progress on track.

The *milestone* concept of Agile methodology aims to first create fully functional software for use with continual updates. For the first milestone, the team developed a web application to retrieve sleep data from the Fitbit devices. The team demonstrated the web application and ran beta tests while developing the second milestone, the mobile application. Through an iterative process, we integrated new ideas and features to tailor the software to the users’ needs.

## Results

### Overall design

**Fig. 1:**
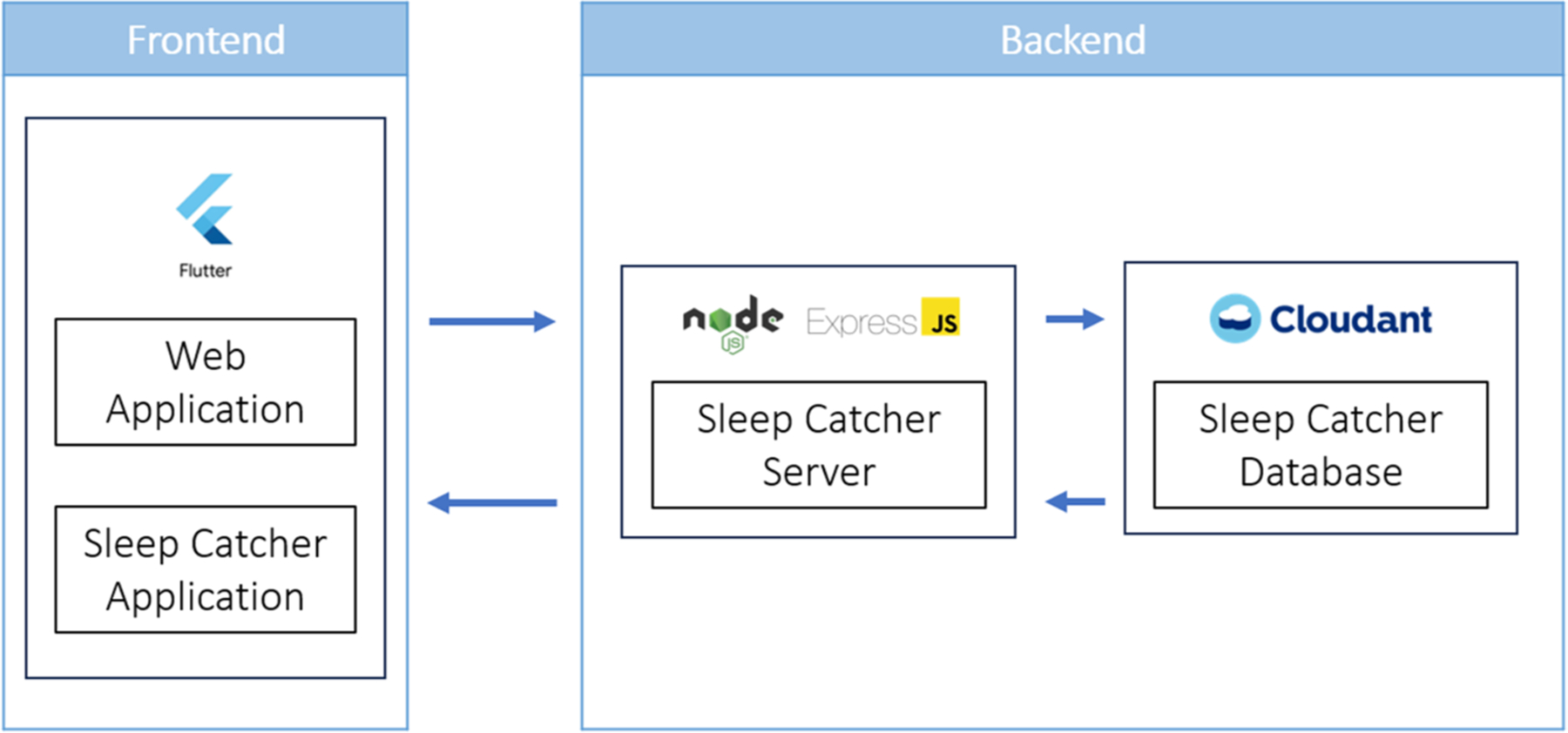
Full stack diagram of the “Sleep Catcher” workflow. The frontend is comprised of the web application and “Sleep Catcher” mobile application, built on Flutter. The backend is comprised of the “Sleep Catcher” server, powered by Node and Express.JS, and a Cloudant database

A full stack system of multiple applications using three-tier software application architecture, which included a web application, a server, and a database, was created. As shown in Fig. 1, Flutter, a cross-platform development framework, was used to build the frontend web and mobile applications. Our server is hosted by IBM Code Engine, a serverless platform used to create containerized applications, and is written with Node.js and Express.js, the two robust and popular choice for server development. IBM Cloudant Database was used as our database.

We designed the applications, server, and database to meet Health Insurance Portability and Accountability Act (HIPAA) standards. Our software does not store any protected health information. An individual’s sleep diaries are associated with a de-identified participant identification number, in compliance with the UCSD Human Research Protection Program (IRB). Furthermore, the transfer of data between the server, applications, and sleep devices will employ the Hypertext Transfer Protocol Secure (HTTPS) protocol which encrypts data to ensure a high level of security. The database is hosted on IBM Cloud and is protected by physical and network security measures.

#### Web application

The web application was implemented before the mobile application, and it serves as a platform for researchers or clinicians to access all data related to each participant including from the Fitbit device, Withings device, and user-entered surveys. During the development of the web application, the engineering team became familiar with the APIs and Flutter. Web applications are lightweight and easy to test and therefore served as the first milestone. In addition, as both the web application and mobile application use Flutter, the web application setup was reused when developing the mobile application, greatly improving the efficiency of mobile application development. The purpose and benefit of the web application became increasingly apparent throughout the project. One common problem with existing CBT-i apps is the lack of clinical integration as most apps do not have a clinician-specific interface to monitor participant’s status and sleep data. Our web application seeks to solve this problem by providing an easy way to access all the device and survey data.

#### Server and database

The server enables two-way communication between the participant-use mobile application and the clinician-use web application, allowing clinicians to get updated status and data from the mobile application easily. The database stores survey-recorded data as well as the Fitbit and Withings API access tokens for each participant. The server periodically requests updated API access tokens to enable continual retrieval of the device data.

**Fig. 2:**
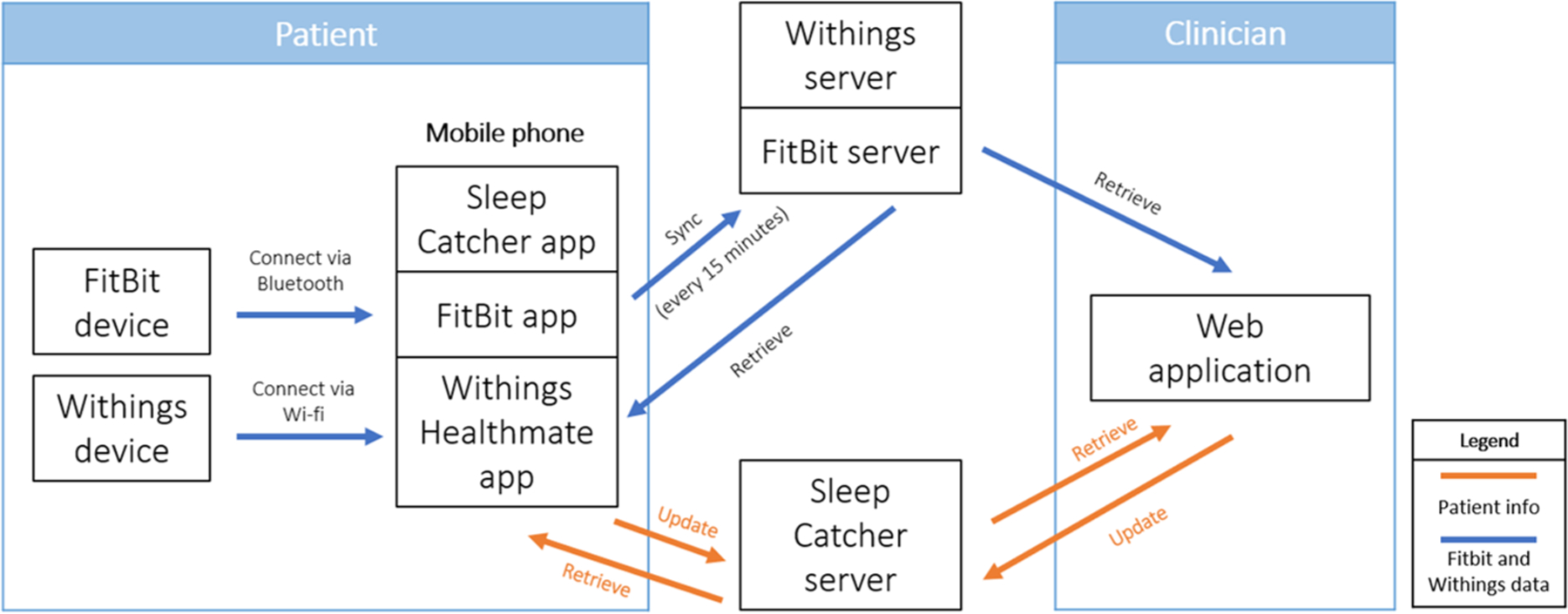
Data flow diagram relating patient’s devices and the clinician’s web application. Patient data is collected through the Fitbit, Withings, and mobile devices, which is fed into their respective servers. The clinician uses the web application to retrieve and update patient data

As shown in Fig. 2, the patient will be using three devices: a Fitbit watch, a Withings sleep mat, and a mobile phone (Android). The sleep tracking devices connect to the phone via Bluetooth and Wi-Fi. The mobile phone connects to the Fitbit server, the Withings server, and our Sleep Catcher server to update and retrieve necessary data. Similarly, a clinician will interact with the web application only and access all patient data collected by the servers.

### Mobile application

#### User Interface (UI) design

User interface is a crucial part of application development and can lead to increased usage and user satisfaction, while failure to do so can steer users away from using an application. In recognition of the high prevalence of cognitive impairments among people with SMI, our team referred to the World Wide Web Consortium (W3C)’s document on improving content usability in people with cognitive disability [17]. The document has detailed criteria for architecture design choices when developing a web application. To summarize the criteria, developers must build applications that help users understand how to use certain features on the application, use concise content, and does not involve processes that rely heavily on memory.

With this framework in mind, our application has five pages ready for the user: Home, CBT-i, Learn, Stats, and Settings. Although each page serves a purpose, a user can only interact with the Home page and still complete the sleep diaries. A traditional sleep diary report requires patients to report and compute various sleep-related variables, as well as answer questions regarding their sleep. Our mobile application divides the sleep diary components (questions about sleep duration, awakenings, quality, etc.) and simplifies data collection by using a set of five flashcards, which appear one at a time.

**Fig. 3:**
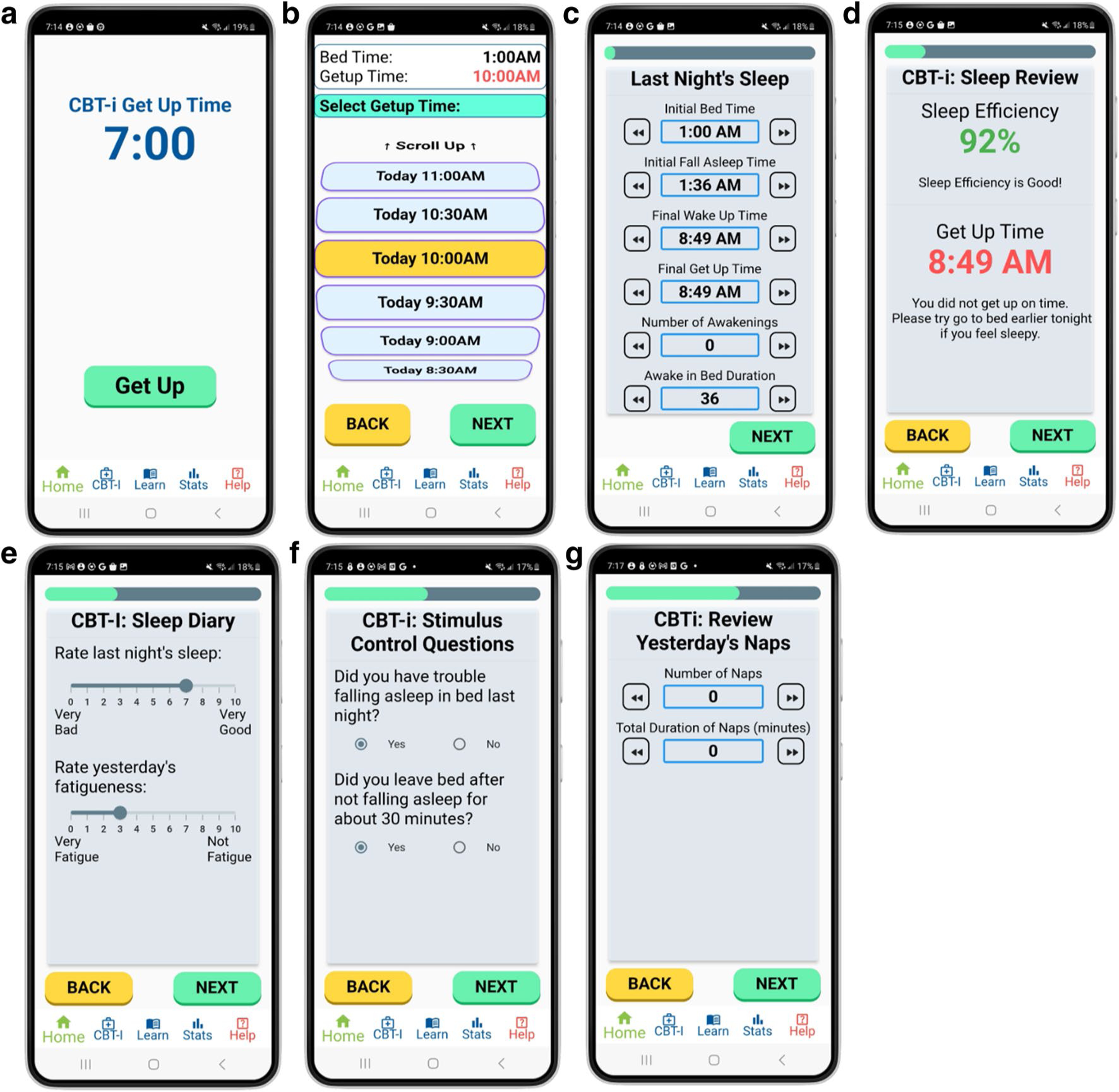
**A–G** Sequential examples of flashcards available to participants when completing their daily sleep diary. The sleep diary card is prefilled with data inputs from the sleep sensors, which participants can adjust. Participants are also asked to provide qualitative answers regarding the previous night’s sleep

To aid users in completing the sleep diaries, several to-do-list-style flashcards were created (shown in Fig. 3A–G). On a given night, the user wears a Fitbit watch and sleeps on a mattress which has a Withings sleep mat under it. When the user wakes up in the morning, they open the “Sleep Catcher” mobile application to complete their sleep diary for the day. The steps of completing the sleep diary are as follows:

1. Tap “Get Up” to start generating the sleep diary (Fig. 3A).
2. Select the time you got into bed (bed-time) and got out of bed (getup time) (Fig. 3B).
3. Review and edit the sleep diary measures generated from the Fitbit and Withings devices (Fig. 3C).
4. Receive feedback on your sleep efficiency and get up time, relative to your targets (Fig. 3D).
5. Rate the quality of last night’s sleep and your fatigue level this morning (Fig. 3E).
6. Answer questions about difficulties falling asleep and getting out of bed (Fig. 3F).
7. Review and update information about the naps from the previous day (Fig. 3G).

We chose to have “next” and “back” buttons to navigate between the flashcards to eliminate the need for swiping, as that may be less intuitive for individuals who have less familiarity with smartphones. The application is only available for English speakers at this time. Concise language and simple icons were used to increase comprehensibility. We simplified the language by writing at a 7th grade level or lower, as recommended by prior work on website design for individuals with cognitive deficits [18]. The Flesch-Kincaid grade level index [19] of texts, buttons, and instructions on the mobile app is 6.7, indicating a 6th grade level of language. The application’s navigation bar includes icons that are indicative of each page’s purpose. Furthermore, to decrease the reliance on the user’s memory and aid users with cognitive deficits, the mobile application uses data from Fitbit and Withings devices to provide estimates of when the user got into bed, fell asleep, etc. (Fig. 3C). Users can review these timestamps and make adjustments if they believe that the reports are inaccurate.

### Testing and continuous delivery

Following the initial development of the application, testing and continuous delivery, a core concept of Agile methodology, were conducted. After the release of each milestone, a series of beta tests were performed under different conditions to gather feedback on the application’s feasibility, stability, and reliability. Using continuous delivery, real-time updates were developed and implemented, which allowed for the incorporation of relevant API and UI design changes.

#### Feasibility studies

The first two beta tests were instrumental in understanding the feasibility of using the application, though the initial aims were to validate sleep log generation from Fitbit and Withings and to obtain feedback on user experience. However, the users had challenges in downloading the application and setting up the devices independently. For the second beta test, a protocol to set up the devices and application prior to the test, as well as set of written instructions for the user, were created. Although the user was able to use the application, there were challenges in providing remote real-time support for the test.

To address this, we made several modifications to the application. Users can now receive live feedback and troubleshooting by contacting the study staff or clinicians. The Help page of the app includes a button that users can click to call the study staff. We also installed commercially available application to mirror and remotely control the smartphone. Users can be guided over the phone call to allow the study staff or clinicians to remotely access the phone and directly troubleshoot any issues with the app or the phone in general. Additionally, the Help page includes a Frequently Asked Questions section to assist with common issues.

#### Device stability study

The third beta test had the aim to validate sleep diary generation from the mobile application. Based on the findings from the previous beta tests, a device introduction period was incorporated into the project timeline. During this 6-day period, the user became familiar with the Fitbit and Withings devices and the research team ensured that the application and devices were collecting the needed data. Once the research team confirmed the proper functioning of the devices and data transfer, the tester then began to complete the application-based sleep diaries at Day 9. This beta test confirmed that the server successfully saved all the sleep diaries entered by the tester. The device introduction period was helpful for both the developers and users. This period also mirrors the initial screening phase of CBT-i treatment to establish baseline sleep characteristics prior to the intervention.

#### Reliability studies

The last set of beta tests focused on improving the device-generated sleep diary entries in a more reliable way. Previously, the user would open the application and was required to wait a period of time for the devices to sync with the application. The developers added an automated functionality for the application to sync with both devices when the user initiated their check-in in the morning. This process ensured the generation of suggested sleep diary entries for the user. These beta tests were successful in generating sleep diary entries in a timely and reliable fashion.

In summary, the beta tests led to changes in the user-level assistance, research protocol, and device-level data syncing to improve usability, stability, and reliability.

#### Ethical design

We designed the mobile application to adhere to ethical standards of accessibility, transparency, informed consent, and data security. The application was designed for use among individuals with cognitive deficits and limited access to technology, with clear language and simple pages. The application collects and processes only the necessary information from Withings and Fitbit devices. For the Fitbit devices, sleep data is collected for sleep diary generation, and heart rate and battery level are processed to improve adherence. For the Withings devices, only sleep data is collected for sleep diary generation. We abide by the terms of service for the third-party applications (Fitbit and Withings applications). For data security, we provide password protection for the smartphone, Fitbit and Withings applications, and have designed the applications, server, and database to meet HIPAA standards (details in “Overall design” section).

## Discussion

Our mobile application development has been a living example of software engineering, or the practice of solving real-world problems through the development of a piece of software. Our team initiated the development with the set goal of creating a digital tool to support CBT-i among people with SMI and cognitive impairments. An iterative approach to building the web application, mobile application, and the backend structure was implemented for this project. The beta tests were crucial for adaptation of the application for real-world use. The application development process relied upon close collaboration between engineering, sleep medicine, and psychiatry—bringing together unique expertise to tailor a product for a specific clinical audience.

Testing the application in real-world contexts highlighted unforeseen challenges in using the application. Prior to the beta tests, the developers added features and complexities to the application to improve accurate and complete data collection. The developers had assumptions about the intuitive nature of the application features, based on their comfort with using a wide range of apps. Following each beta test, components were removed or simplified from the application to improve practicality and accessibility for users who have less familiarity with smartphones and digital applications. The test users’ feedback provided different perspectives with a wide range of tech literacy. The beta test process was invaluable for the developers to consider the breadth of user proficiency with technology and the adaptation of apps to different environments.

Second, application development relied heavily on multi-disciplinary collaboration between engineering (JMJ, JM, BD, IB), sleep medicine (SAI, AM), and psychiatry (PK, EEL). The application developers (JMJ, JM) worked with the co-authors to gain perspective on the needs of the target population and to understand how to adapt CBT-i sleep diaries to a digital format. Through shadowing research assessments with participants with SMI, the developers gained insights into the availability and familiarity of technology among the target population. Through consultation with the sleep experts, the developers focused their application on simplified set of guidelines to improve feasibility and usage in a population with cognitive deficits and health challenges. The iterative process enabled continual refinement and simplification of the application for the target population. The collaboration also generated new algorithms for analyzing wearable and environmental sleep sensor data within this population, with potential to improve established research and clinical processes.

Last, digital health disparities among people with SMI were considered while developing the mobile application. Relative to the general population, individuals with SMI are less likely to use technology [20, 21], due in part to financial limitations, difficulties in accessing and navigating technology [22], and disease-related factors (e.g., cognitive deficits, less familiarity with the internet and computers) [23, 24]. Additional reasons for hesitancy in using technology include paranoia associated with computer and email use, psychosis-related concentration difficulty, stimulus overflow, and fear of symptom provocation [20, 22]. Several commercially available CBT-i apps were found to strictly exclude people with mental illnesses—which may reflect the lack of targeted development and testing of these apps for this population. Furthermore, many applications intended for use in people with SMI have not adopted a user-centered approach during application development [25]. While there are notable barriers to access, digital health technologies show promise in helping people with SMI seek out information and social interaction without fear of stigma [22]. Individuals with SMI who can successfully utilize technology have been able to identify coping strategies and set reminders for medication management, indicating the promise of technology’s benefits on illness management and recovery [26]. Further work is needed to elevate digital literacy among people with SMI and to design applications with the input of people with lived experiences.

Traditionally sleep diaries are based on subjective impressions of sleep, as objective data may differ. For example, subjective and objective sleep duration may be discordant in some cases, particularly among people with insomnia. This dissociation used to be labeled sleep-state misperception but is now more commonly referred to as paradoxical insomnia in which patients report being awake all night even when objective data suggest otherwise. Paradoxical insomnia may be particularly common in psychiatric disorders, although data in this context remain sparse. Our novel mobile application is hoped to facilitate further research in this context and help to design and optimize therapeutic strategies. While this application was designed to use objective measures to aid in capturing subjective impressions of sleep, further work is needed to understand the utility of these sleep diaries for people with SMI.

Moving forward, our group is preparing to obtain user feedback from individuals with SMI on the design and usability of the application. Next steps include focus groups and individual feedback sessions on the design and purpose of the application to elicit specific feedback on the user interface. After incorporating these suggestions into the application, we plan to conduct beta tests among people with SMI. We are assessing our study cohort’s access to Wi-Fi Internet services and comfort with using smartphones and apps as potential barriers to the broad use of such applications. These feasibility tests will help to reveal how this application can help people with SMI and whether it supports the successful implementation of CBT-i.

To design and develop a mobile application for a clinical population’s specific needs, adopting an iterative and innovative process such as the agile methodology is critical. Collaboration between engineering, psychiatry, and sleep medicine has led to incremental improvements and further refinement of the application’s purpose and function. The joint effort not only led to the creation of a novel application to help people with SMI track their sleep, but also enabled better understanding of the clinical needs for developers and the engineering challenges for the clinicians. Future work includes seeking feedback from people with SMI, our target demographic group, with the aim of improving mobile application user interface and user experience. These adjustments will help us reach the ultimate goal of supporting CBT-i for individuals with SMI.

## Data Availability

This manuscript has no associated data.

## References

[1] Seow LSE (2018) “Evaluating DSM-5 insomnia disorder and the treatment of sleep problems in a psychiatric population”, (in eng). J Clin Sleep Med 14(2):237–244. 10.5664/jcsm.6942 29394962 PMC5786843

[2] Baglioni C, Spiegelhalder K, Lombardo C, Riemann D (2010) Sleep and emotions: a focus on insomnia. Sleep Med Rev 14(4):227–238. 10.1016/j.smrv.2009.10.007 20137989

[3] Irwin MR, Carrillo C, Sadeghi N, Bjurstrom MF, Breen EC, Olmstead R (2022) Prevention of incident and recurrent major depression in older adults with insomnia: a randomized clinical trial. JAMA Psychiat 79(1):33–41. 10.1001/jamapsychiatry.2021.3422 PMC8733847 34817561

[4] Mausbach BT (2010) “Relationship of the Brief UCSD Performance-based Skills Assessment (UPSA-B) to multiple indicators of functioning in people with schizophrenia and bipolar disorder”, (in eng). Bipolar Disord 12(1):45–55. 10.1111/j.1399-5618.2009.00787.x 20148866 PMC2846793

[5] Harvey AG (2015) Treating insomnia improves mood state, sleep, and functioning in bipolar disorder: a pilot randomized controlled trial. J Consult Clin Psychol 83(3):564–577. 10.1037/a0038655 25622197 PMC4446240

[6] Hertenstein E (2022) Cognitive behavioral therapy for insomnia in patients with mental disorders and comorbid insomnia: a systematic review and meta-analysis. Sleep Med Rev 62:101597. 10.1016/j.smrv.2022.101597 35240417

[7] Krystal AD, Prather AA, Ashbrook LH (2019) The assessment and management of insomnia: an update. World Psychiatry 18(3):337–352. 10.1002/wps.20674 31496087 PMC6732697

[8] Hwang DK, Nam M, Lee YG (2019) “The effect of cognitive behavioral therapy for insomnia in schizophrenia patients with sleep disturbance: a non-randomized, assessor-blind trial”, (in eng). Psychiatry Res 274:182–188. 10.1016/j.psychres.2019.02.002 30807969

[9] Waters F, Chiu VW, Dragovic M, Ree M (2020) “Different patterns of treatment response to Cognitive-Behavioural Therapy for Insomnia (CBT-I) in psychosis”, (in eng). Schizophr Res 221:57–62. 10.1016/j.schres.2020.03.054 32317223

[10] Sivertsen B (2006) Cognitive behavioral therapy vs zopiclone for treatment of chronic primary insomnia in older adults: a randomized controlled trial. JAMA 295(24):2851–2858. 10.1001/jama.295.24.2851 16804151

[11] Freeman D (2015) “Efficacy of cognitive behavioural therapy for sleep improvement in patients with persistent delusions and hallucinations (BEST): a prospective, assessor-blind, randomised controlled pilot trial”, (in eng). Lancet Psychiatry 2(11):975–983. 10.1016/s2215-0366(15)00314-4 26363701 PMC4641164

[12] Walker J, Muench A, Perlis ML, Vargas I (2022) Cognitive Behavioral Therapy for Insomnia (CBT-I): a primer. Klin Spec Psihol 11(2):123–137. 10.17759/cpse.2022110208 36908717 PMC10002474

[13] Guo JY, Ragland JD, Carter CS (2019) “Memory and cognition in schizophrenia”, (in eng). Mol Psychiatry 24(5):633–642. 10.1038/s41380-018-0231-1 30242229 PMC6428626

[14] Troxel WM, Germain A, Buysse DJ (2012) Clinical management of insomnia with brief behavioral treatment (BBTI). Behav Sleep Med 10(4):266–279. 10.1080/15402002.2011.607200 22946736 PMC3622949

[15] Beattie Z, Pantelopoulos A, Ghoreyshi A, Oyang Y, Statan A, Heneghan C (2017) 0068 estimation of sleep stages using cardiac and accelerometer data from a wrist-worn device. Sleep 40(suppl_1):A26–A26. 10.1093/sleepj/zsx050.067 29087960

[16] Edouard P (2021) Validation of the Withings Sleep Analyzer, an under-the-mattress device for the detection of moderatesevere sleep apnea syndrome. J Clin Sleep Med 17(6):1217–1227. 10.5664/jcsm.9168 33590821 PMC8314651

[17] (2021) Making Content Usable for People with Cognitive and Learning Disabilities, W3C Working Group Note W. W. W. Consortium, April 29, 2021. [Online]. Available: https://www.w3.org/TR/coga-usable/. Accessed 19 May 2023

[18] Rotondi AJ (2007) “Designing websites for persons with cognitive deficits: design and usability of a psychoeducational intervention for persons with severe mental illness”, (in eng). Psychol Serv 4(3):202–224. 10.1037/1541-1559.4.3.202 26321884 PMC4552350

[19] Kincaid JP, Fishburne R Jr, Robert P, Richard L, Brad S (1975) Derivation of new readability formulas (automated readability index, fog count and flesch reading ease formula) for navy enlisted personnel. Defense Technical Information Center, 1975/02/01. [Online]. Available: 10.21236/ada006655

[20] Miller BJ, Stewart A, Schrimsher J, Peeples D, Buckley PF (2015) How connected are people with schizophrenia? Cell phone, computer, email, and social media use. Psychiatry Res 225(3):458–463. 10.1016/j.psychres.2014.11.067 25563669

[21] Ben-Zeev D, Davis KE, Kaiser S, Krzsos I, Drake RE (2013) Mobile technologies among people with serious mental illness: opportunities for future services. Adm Policy Ment Health 40(4):340–343. 10.1007/s10488-012-0424-x 22648635 PMC4007758

[22] Schrank B, Sibitz I, Unger A, Amering M (2010) How patients with schizophrenia use the internet: qualitative study. J Med Internet Res 12(5):e70. 10.2196/jmir.1550 21169176 PMC3057320

[23] Depp CA (2010) Mobile interventions for severe mental illness: design and preliminary data from three approaches. J Nerv Ment Dis 198(10):715–721. 10.1097/NMD.0b013e3181f49ea3 20921861 PMC3215591

[24] Robotham D, Satkunanathan S, Doughty L, Wykes T (2016) Do we still have a digital divide in mental health? A five-year survey follow-up. J Med Internet Res 18(11):e309. 10.2196/jmir.6511 27876684 PMC5141335

[25] Batra S, Baker RA, Wang T, Forma F, DiBiasi F, Peters-Strickland T (2017) Digital health technology for use in patients with serious mental illness: a systematic review of the literature. Med Devices (Auckl) 10:237–251. 10.2147/MDER.S144158 29042823 PMC5633292

[26] Gay K, Torous J, Joseph A, Pandya A, Duckworth K (2016) Digital technology use among individuals with schizophrenia: results of an online survey. JMIR Ment Health 3(2):e15. 10.2196/mental.5379 27146094 PMC4871994

